# Shotgun Lipidomics Revealed Altered Profiles of Serum Lipids in Systemic Lupus Erythematosus Closely Associated with Disease Activity

**DOI:** 10.3390/biom8040105

**Published:** 2018-10-03

**Authors:** Lu Lu, Changfeng Hu, Yanxia Zhao, Lijiao He, Jia Zhou, Haichang Li, Yu Du, Yonghua Wang, Chengping Wen, Xianlin Han, Yongsheng Fan

**Affiliations:** 1 College of Pharmaceutical Sciences, Zhejiang Chinese Medical University, 548# Bingwen Road, Hangzhou, Zhejiang 310053, China; luluhu1990@163.com; 2 College of Basic Medical Sciences, Zhejiang Chinese Medical University, Hangzhou, Zhejiang 310053, China; zhudianzhuifeng@163.com (C.H.); 13067765539@163.com (Y.Z.); 15912378428@sina.cn (L.H.); zhoujia_1990@163.com (J.Z.); lihaichango@163.com (H.L.); duyuzjtcm@163.com (Y.D.); cpwen.zcmu@yahoo.com (C.W.); 3 School of Food Science and Engineering, South China University of Technology, Guangzhou 510640, China; yonghw@scut.edu.cn; 4 Barshop Institute for Longevity and Aging Studies, University of Texas Health San Antonio, 7703 Floyd Curl Drive, Mail Code 8254, San Antonio, TX 78229-3900, USA; 5 Department of Medicine-Diabetes, University of Texas Health San Antonio, San Antonio, TX 78229-3900, USA

**Keywords:** systemic lupus erythematosus, lipid metabolism, serum lipidome, shotgun lipidomics, ceramide, phosphatidylethanolamine

## Abstract

The pathogenesis of systemic lupus erythematosus (SLE) remains elusive. It appears that serum lipid metabolism is aberrant in SLE patients. Determination of lipid profiles in the serum of SLE patients may provide insights into the underlying mechanism(s) leading to SLE and may discover potential biomarkers for early diagnosis of SLE. This study aimed to identify and quantify the profile of serum lipids in SLE patients (*N* = 30) with our powerful multi-dimensional mass spectrometry-based shotgun lipidomics platform. Multivariate analysis in the form of partial least squares-discriminate analysis was performed, and the associations between the changed lipids with cytokines and SLE disease activity index (SLEDAI) were analyzed using a multiple regression method. The results of this study indicated that the composition of lipid species including diacyl phosphatidylethanolamine (dPE) (16:0/18:2, 18:0/18:2, 16:0/22:6, 18:0/20:4, and 18:0/22:6), 18:2 lysoPC (LPC), and ceramide (N22:0 and N24:1) was significantly altered in SLE patients with *p* < 0.05 and variable importance of the projection (VIP) > 1 in partial least squares-discriminate analysis (PLS-DA). There existed significant associations between IL-10, and both 18:0/18:2 and 16:0/22:6 dPE species with *p* < 0.0001 and predicting 85.7 and 95.8% of the variability of IL-10 levels, respectively. All the altered lipid species could obviously predict IL-10 levels with F (8, 21) = 3.729, *p* = 0.007, and R^2^ = 0.766. There was also a significant correlation between the SLEDAI score and 18:0/18:2 dPE (*p* = 0.031) with explaining 22.6% of the variability of SLEDAI score. Therefore, the panel of changed compositions of dPE and ceramide species may serve as additional biomarkers for early diagnosis and/or prognosis of SLE.

## 1. Introduction

Systemic lupus erythematosus (SLE) is an inflammatory autoimmune disease that is characterized by the immune system attacking self healthy cells and tissues throughout the body, and mainly occurs in women of childbearing age [1]. It can affect multiple major organ systems, such as the heart, blood vessels, the liver, kidneys, and nervous systems and show different manifestations [2]. Although the genetic, environmental, hormonal, and immunoregulatory factors are generally considered to implicate in the expression of tissue injury and the diverse clinical manifestations of SLE patients, the exact pathological mechanisms of SLE have not been understood. Moreover, there is no cure or even good treatment for the disease [3]. Therefore, the development of diagnostic tools and effective therapeutics is desirable.

Lipid metabolism is markedly altered in SLE patients. Lipids play key roles in many biological processes, such as cellular barriers, energy storage and metabolism, signaling, and growth and survival [4]. A “lupus pattern” of dyslipoproteinemia, which may be responsible for the development of premature atherosclerosis, has been identified in SLE patients with 7- to 50-fold increased age-specific incidence of cardiovascular disease [5]. Intriguingly, this pattern was also noted in both untreated pediatric and adult patients and further aggravated by disease activity, indicating SLE itself promotes a proatherogenic lipid profile [5,6]. 

Autoantibodies lead to the aberrant metabolism of lipids in SLE patients. So far, ~180 different autoantibodies have been described in patients with SLE [7], although it has not been determined whether all the characteristic autoantibodies are pathogenic in SLE. The targets of these autoantibodies include plasma proteins, matrix proteins, cell membranes, nucleosome, DNA, blood cell, nervous system, etc. [7]. Moreover, some of them are directly involved in lipid metabolism, or target lipids and/or their metabolites, such as antibodies to anionic phospholipids (i.e., cardiolipin, phosphatidylinositol (PI), phosphatidylserine, and phosphatidic acid), antibodies to zwitterionic phospholipids (e.g., phosphatidylethanolamine (PE), phosphatidylcholine (PC), and platelet activating factor), autoantibodies to lipoprotein lipase, and so on [8,9]. These autoantibodies seriously disrupt the metabolic pathways of lipids in SLE patients.

Increased oxidative stress also results in aberrant metabolism of lipids/lipid complexes in SLE patients. According to the literature, oxidative stress is elevated in lupus patients mainly due to mitochondrial dysfunction in abnormal immunocytes [10,11]. It not only increases the levels of proinflammatory cytokines and oxidized low-density lipoprotein (ox-LDL) [12], but also impairs the functions of high-density lipoprotein and induces autoimmune reaction via oxidative modification of self-antigens, which is thought to underlie SLE pathology [13,14]. Therefore, determination of the altered lipid profiles in serum allows us to understand the mechanism(s) underpinning SLE pathogenesis and discover potential biomarkers as novel diagnostic tools.

Although a variety of metabolomics studies on SLE using gas chromatography–mass spectrometry (GC–MS), liquid chromatography–mass spectrometry (LC-MS), or nuclear magnetic resonance (NMR) technology have been published recently, there is no research specially focused on alterations of lipid species [15,16,17,18,19]. In our previous paper, we focused on the changes of lipids related with oxidative stress (including plasmalogens, lysophospholipids, and 4-hydroxy-2(*E*)-nonenal (HNE) species), which were identified and quantified using our multi-dimensional mass spectrometry-based shotgun lipidomics (MDMS-SL) platform [20]. In the study, we revealed significant correlations among the SLE disease activity index (SLEDAI), proinflammatory cytokine IL-10, and the levels of ethanolamine plasmalogen (pPE) species for the first time [20]. However, alterations (e.g., level/profile) of other vital lipids (such as PI, lysoPC (LPC), and sphingolipid species) in SLE patients may also provide additional possibilities to discover biomarkers. In the study, other lipids in the same serum samples from 30 SLE patients and 30 controls were further determined by our MDMS-SL platform. The concentrations/profiles of these lipids were compared using multivariate and multiple regression analyses in attempting to discover novel alterations in lipid metabolism and new biomarkers for early diagnosis of SLE in addition to our previously uncovered peroxidation-mediated lipid species.

## 2. Materials and Methods

### 2.1. Patients

As previously published [20], the patients were selected from more than 300 lupus patients, who meet at least 4 of the revised American College of Rheumatology Classification criteria for SLE [21]. The controls were healthy with no clinical manifestations of SLE. All individuals were female between ages of 20 and 55, and none of them were pregnant at the time of study. Moreover, all of the individuals never smoke or smoke rarely. The study was conducted in accordance with the Declaration of Helsinki and the protocol was approved by the Ethic Committee of Zhejiang Chinese Medical University (project ID: No. 2013zjtcm-016; date of approval: 16 February 2013). Written informed consents were obtained from all participating people. In addition, the patients were asked not to take any medicine for at least 24 h before drawing blood.

### 2.2. Blood Collection

All patients and controls were asked to eat a balanced diet (they should have adequate amounts of fresh fruits and vegetables in addition to keeping their normal food habits) for at least 1 week before drawing blood. Blood samples were collected in early morning after the participants fasted for at least 12 h. Sera were obtained through centrifugal separation and then stored at −80 °C prior to use. According to manufacturer’s instructions, the levels of cytokine IL-10 in each serum sample were measured through human cytokine detection kits with 500 µL of serum sample (Multisciences Corporation, Hangzhou, China).

### 2.3. Lipid Standards and Chemicals

All lipid internal standards were obtained from Avanti Polar lipids, Inc. (Alabaster, AL, USA), Nu Chek, Inc. (Elysian, MN, USA), or Matreya, Inc. (Pleasant Gap, PA, USA). All of the solvents were purchased from Burdick and Jackson (Honeywell International Inc., Muskegon, MI, USA). All other reagents were at least of analytical reagent grade and obtained from Sigma-Aldrich Chemical Company (St. Louis, MO, USA), Fisher Scientific (Pittsburgh, PA, USA), or indicated. 

### 2.4. Preparation of Lipid Extracts from Serum Samples

Lipid extracts were prepared from individual samples according to a modified protocol of Bligh and Dyer extraction [22] in the presence of internal standards as previously described [23]. Briefly, a premixed solution including all kinds of internal standards for quantification of lipid species was added into each serum sample (100 µL). After subjecting the mixture to a modified procedure of Bligh-Dye extraction, the chloroform phase was collected and evaporated under a nitrogen stream to obtain individual lipid extract. Each lipid extract was resuspended with 2 µL of chloroform/methanol (1:1 by volume)/µL serum, capped, and then stored at −20 °C for MS analysis as previously described [24].

### 2.5. Mass Spectrometric Analysis of Lipids

A triple-quadrupole mass spectrometer (Thermo TSQ Quantiva, San Jose, CA, USA), equipped with an automated nanospray ion source (TriVersaNanoMate, Advion Bioscience Ltd., Ithaca, NY, USA) and Xcalibur system software, was used in the study [25]. Prior to direct infusion into the mass spectrometer through the NanoMate device, each lipid extract was further diluted to <50 pmol of total lipids/μL with a mixture of chloroform/methanol/isopropanol (1/2/4 by volume). 

Identification and quantification of different lipids were conducted in the study as previously described [24,26]. It is well known that after collision-induced dissociation, the majority of lipid classes have a unique fragment pattern that can be used to determine the structures of these lipid classes. These characteristic properties of lipids were maximally exploited to identify and quantify lipid species of interest by MDMS-SL technology. Briefly, identification of individual lipid species was achieved with the following steps. First, each lipid class or subclass was selectively ionized through intrasource separation. Second, a full MS scan detecting the precursor ions corresponding to the molecular species of a class of interest was specifically acquired in an appropriated mass region. Finally, a multiple tandem MS scan (e.g., precursor ion scan and neutral loss scans) related to the lipid class of interest were acquired in the same mass regions. The full MS scan and the set of tandem MS scan were used to construct a 2D mass spectrum. Each individual lipid species, including fatty acyl chains, isomeric and isobaric species, and regiospecific positions was identified through analysis of these cross peaks in 2D mass maps. More detailed information on identification and quantification of different lipids could consult the book entitled *Lipidomics: Comprehensive Mass Spectrometry of Lipids* [27].

In MS analysis, the first and third quadrupoles were utilized as independent mass analyzers with a mass resolution setting of 0.7 Th, and the second quadrupole was used as a collision cell for tandem MS analysis. Both the full scan mass and the tandem MS spectra were automatically acquired by a custom sequence subroutine operated under Xcalibur software. Usually, a 1- to 2-min or 2- to 5-min period of signal averaging in the profile mode was used for each full MS scan or tandem MS analysis, respectively. In addition, the collision gas pressure was set at 1.0 mtorr, and the collision energy varied according to the classes of lipids for tandem MS analysis [24]. 

### 2.6. Data Processing

MS data processing, including baseline correction, ^13^C de-isotoping, peak intensity comparison, and quantitation, was conducted with custom-programmed Microsoft Excel Macros [24]. The levels of lipid species were normalized to the serum volume. All data were presented as mean ± standard error of the mean (SEM) or the composition of individual lipid species in the class unless otherwise indicated. Student’s unpaired *t* tests were employed to determine significant differences between the groups. To further compare the lipid profile of different people, the compositions of lipid species were subjected to multivariate analysis in the form of PLS-DA using SIMCA-P+ (V11.0.0.0 Umetrics AB, Umea, Sweden). In order to determine the association among changed lipid species, inflammation, and the severity of SLE, optimal curve correlation of individual lipid species and multiple regression analysis of the panel of all the changed lipid species with IL-10 or SLEDAI were performed using SPSS (19.0) [13]. A value of *p* < 0.05 was considered statistically significant.

## 3. Results

### 3.1. Basic Characteristics of the Subjects

As previously described [20], the study recruited 30 SLE patients and 30 controls with matched age and body mass index. Most of the patients were in the inactive period with SLEDAI ≤ 11 (shown in Table 1). All patients received standard treatment according to their disease activities and other criteria. None of the individuals had taken antioxidants, lipid-lowing agents, and related medicine in the last three months.

### 3.2. Lipidomics Analysis of Phospholipids of Serum Lipid Extracts

Phospholipids, constituting the main components of cell membranes, play a wide variety of roles within organisms, including cell signaling, protein modification, membrane anchoring, and reservoirs for signaling molecules [28,29]. Phospholipids in plasma/serum are abundant and heterogeneous, and distributed among all lipoprotein classes. 

**Figure 1:**
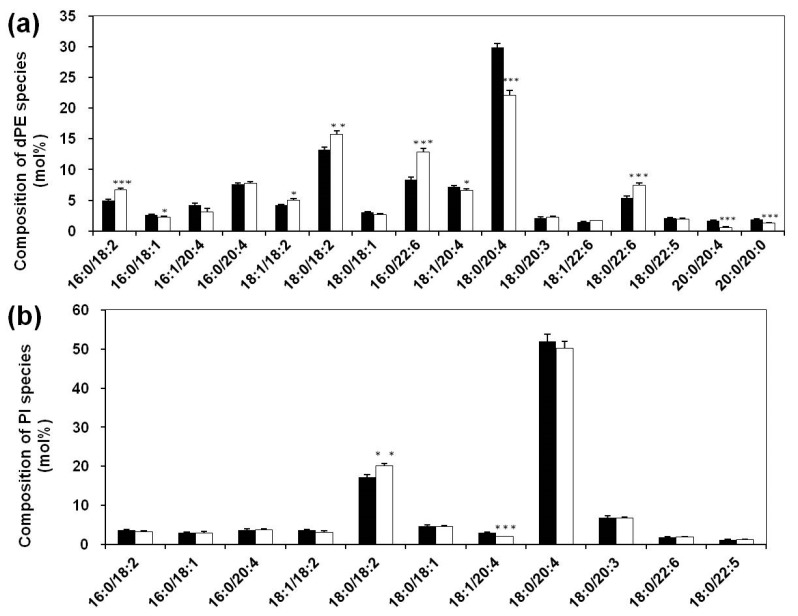
Comparison of the compositions of phosphatidylethanolamine (dPE) and phosphatidylinositol (PI) species present in serum between systemic lupus erythematosus (SLE) patients and healthy controls. Serum lipid extracts from SLE (*N* = 30, open bar) and control (*N* = 30, solid bar) groups were prepared by using a modified Bligh and Dyer extraction protocol as described in Section 2. The data represented mean ± standard error of mean (SEM) (*N* = 30 per group) from different peoples. * p < 0.05, ** p < 0.01, *** p < 0.001 compared with controls.

As revealed previously, the elevated oxidative stress resulted in substantial reduction of plasmalogen, including plasmenylcholine (pPC) and pPE, with the total losses of ~26 and ~21 mol%, respectively [20]. Although the levels of the other subclass of PE (i.e., dPE and plasmanylethanolamine (aPE)) and PC (e.g., diacly-phosphatidylcholine (dPC) and plasmanylcholine) in SLE patients did not change significantly [20], there was a significant difference between the profiles of dPE species of serum from patients and controls (Figure 1a). Compared to the controls, the most increased dPE species in SLE patients were 16:0/18:2 (from 5.00 ± 0.21 to 6.70 ± 0.26 mol%), 18:0/18:2 (from 13.28 ± 0.37 to 15.68 ± 0.60 mol%), 16:0/22:6 (from 8.33 ± 0.42 to 12.80 ± 0.65 mol%), and 18:0/22:6 (from 5.40 ± 0.25 to 7.48 ± 0.37 mol%) with ~34%, 18%, 54%, and 39% elevated, respectively (*p* < 0.001). Intriguingly, almost all compositions of dPE species containing 20:4 fatty acyl chain at the *sn*-2 position appeared reduced to a certain degree, such as 18:0/20:4 (29.86 ± 0.67 and 22.10 ± 0.67 mol% in control and patients, respectively, a reduction of ~26%), and 20:0/20:4 (1.70 ± 0.10 and 0.57 ± 0.11 mol% in control and patients, respectively, a reduction of ~66%) (*p* < 0.001).

In the study, the profile of dPC species between the two groups was also compared. It was found that the majority of their compositions in sera of SLE patients remained unaltered in comparison to those in the control group (results were not shown). In addition, the compositions of LPC species in sera of control patients were also compared. The compositions of almost all LPC species were not changed, except 18:2 LPC. Its composition reduced from 22.47 ± 0.73 in controls to 19.97 ± 0.49 mol% in SLE patients (a reduction of ~11%, *p* < 0.01). 

PI and its derivatives regulate many vital cell processes, such as membrane dynamics, fusion, and trafficking, as well as serving as precursors of second messengers in signal transduction [30]. Recent evidence indicates that PI involves Toll-like receptor signaling, which is very important for the innate immune response and is implicated in diverse pathophysiological disorders [31]. Therefore, the metabolism of PI species in serum of SLE patients could be characteristic and representative since SLE pathogenesis is largely due to dysfunction of immunocytes. MDMS-SL analysis results indicated that the total amount of PI species did not change obviously (21.45 ± 0.95 and 20.35 ± 0.94 nmol/mL in controls and patients, respectively, *p* = 0.42). Although the levels of PI species were not altered, the composition of 18:0/18:2 PI, which is one of the most predominant PI species and accounts for approximately 18% of the total PI amounts in the serum, increased from 17.26 ± 0.69 in controls to 20.07 ± 0.71 mol% in SLE patients (an increment of ~16%, *p* < 0.01) (Figure 1b). As with dPE species, the compositions of PI species containing 20:4 FA chain at the *sn*-2 position appeared reduced in SLE patients to different degrees, especially 18:1/20:4 PI (*p* < 0.001) compared with the controls. 

### 3.3. Altered Composition of Sphingolipid Species in Sera of Systemic Lupus Erythematosus Patients

**Figure 2:**
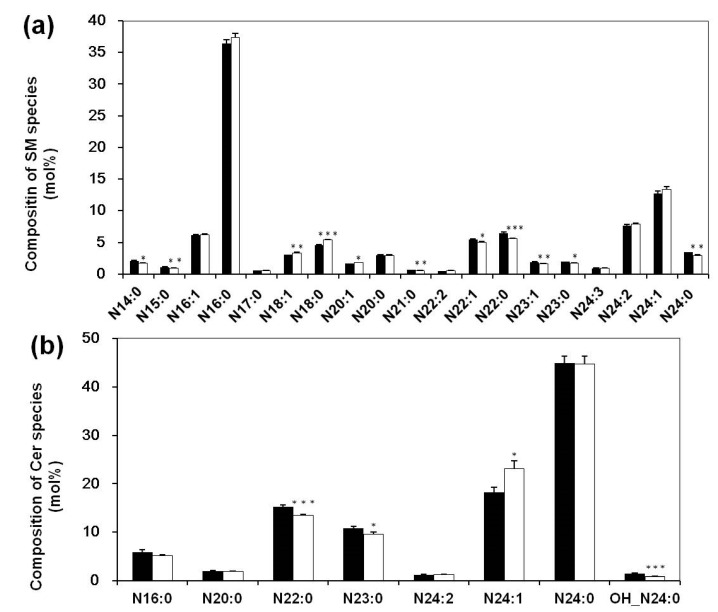
Comparison of the compositions of sphingomyelin (SM) and ceramide (Cer) species present in serum between SLE patients and healthy controls. The serum lipid extracts from SLE (*N* = 30, open bar) and control (*N* = 30, solid bar) groups were prepared by using a modified Bligh and Dyer extraction protocol as described in Section 2. The data represented mean ± SEM (*N* = 30 per group) from different peoples. * p < 0.05, ** p < 0.01, *** p < 0.001 compared with controls. “N” represents the amide linage of the acyl chain.

Besides as essential components of cellular membranes and lipoproteins in organisms, sphingolipid metabolites are signaling molecules involving a diverse range of cellular processes that are important in immunity and inflammation [32]. Moreover, accumulating evidence indicates that sphingolipids (including sphingomyelin (SM) and ceramide (Cer)) with different acyl chain lengths play distinct pathophysiological roles in disease models [33]. Therefore, SM and Cer were identified and quantified in the study to characterize the metabolism of sphingolipids in SLE patients. MDMS-SL analysis demonstrated that the total content of SM species was not changed in SLE patients in comparison to the control (173.81 ± 5.45 and 184.83 ± 8.01 nmol/mL in controls and patients, respectively, *p* = 0.25). Moreover, the contents of the majority of predominant SM species were not significantly different between the two groups, except that SM species comprised with C18 fatty acyl, such as N18:1 and N18:0 SMs. Compared with the controls, the contents of these species elevated in SLE patients to a different degree (increments of ~18 mol% and 25 mol% with *p* < 0.01 and *p* < 0.001, respectively). To further understand the aberrant metabolism of SM species, the profile of SM species in serum was also compared between two groups. In contrast to the unchanged contents of the majority of SMs, there were obviously different compositions of SMs between the controls and SLE patients (Figure 2a).

Cer species serve as central components in sphingolipid metabolism and catabolism [34,35], so the determination of their levels and compositions would contribute to explain the alteration in the compositions of SM species. Like SM species, the levels of different Cer species did not change significantly, but the profile of Cer species had been modified (Figure 2b), especially those containing very-long fatty acyl chains (C22–C24). Specifically, the compositions of N22:0 and N23:0 Cer species (“N” stands for the amide linage of the acyl chain) reduced from 15.27 ± 0.33 and 10.81 ± 0.39 in controls to 13.47 ± 0.28 and 9.61 ± 0.40 mol% in SLE patients, respectively (a reduction of ~10 % with *p* < 0.001 and *p* < 0.05, respectively), while the composition of N24:1 Cer, the second abundant Cer species in serum, increased significantly (18.25 ± 1.03 and 23.08 ± 1.68 mol% in controls and patients, respectively, ~26% elevated, *p* < 0.05). Additionally, the composition of N24:0 Cer with a hydroxyl group on the second position of the acyl chain also showed a reduction from 1.53 ± 0.11 in controls to 0.88 ± 0.1 mol% in patients (*p* < 0.001). 

### 3.4. Multivariate Analysis of Lipid Profiles of Different Groups

**Figure 3:**
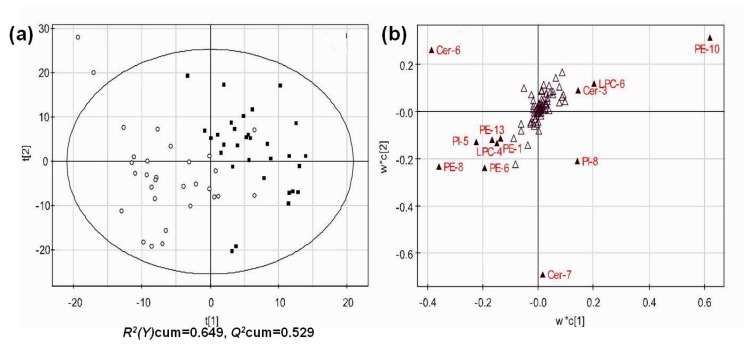
Multivariate analysis of lipid profile. The partial least squares-discriminate analysis (PLS-DA) score (**a**) and loading (**b**) plots were obtained based on the profiles of different lipid classes in sera from different people of SLE (*N* = 30, open circles) and normal (*N* = 30, solid squares). Figure 3b shows the variable importance of the projection (VIP) plot, indicting which variables are important in explaining both the X- and Y-data. The solid triangles (VIP value > 1.0) in Figure 3b are important contributors to the model. The detailed information about abbreviation of lipid species is shown in Table S1.

To get an overview of the lipid profile of different people and find which variables contribute to the separation of two groups, partial least squares-discriminate analysis (PLS-DA) was performed and a score plot was obtained with *R*^2^(*Y*) value of 0.649 and a good prediction parameter *Q*^2^ = 0.529 (Figure 3). The PLS-DA model was validated through permutation test to avoid overfitting. The score plot showed that there was an obvious clustering trend between SLE and normal groups based on the compositions of 83 species of LPC, dPC, dPE, PI, Cer, and SM in serum, suggesting notably intrinsic differences between two groups. The lipid species with variable importance of the projection (VIP) > 1 were regarded as main contributors to the classification of the two groups and marked with red color in the loading plot (Figure 3b). These species with *p* < 0.05 and VIP > 1 included dPE (16:0/18:2, 18:0/18:2, 16:0/22:6, 18:0/20:4, and 18:0/22:6), 18:2 LPC, and Cer (N22:0, and N24:1). These lipid species were further associated with SLEDAI and proinflammatory cytokines.

### 3.5. Significant Association of IL-10 with the Compositions of the Altered Lipids

**Figure 4:**
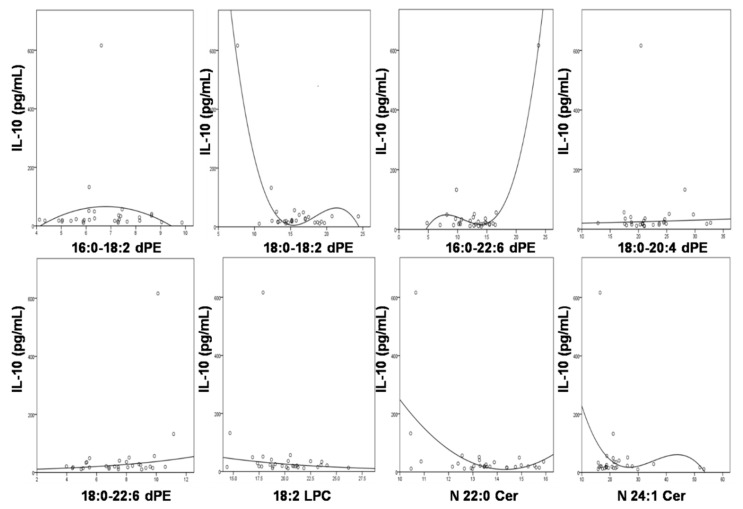
Optimal curve fitting between the levels of cytokine IL-10 and the compositions of those significantly altered lipid species in sera of SLE patients. The unit of the x-axis is “%”.

As the published findings, cytokine IL-10 was positively associated with many clinical indices, especially central nervous system manifestations [36]. In the study, the relationships of IL-10 levels with the compositions of the changed lipid species were determined (Figure 4). There existed significant associations between IL-10 and 18:0/18:2, and 16:0/22:6 dPE species with *p* < 0.0001. These dPE species could predict 85.7 and 95.8% of the variability of IL-10 levels, respectively. Additionally, a multiple regression of a panel of these lipid species was conducted to predict IL-10 levels in SLE patients. It was found that these lipids obviously predicted IL-10 levels with F(8, 21) = 3.729, *p* = 0.007, and R^2^ = 0.766.

### 3.6. Correlation of SLE Disease Activity Index with the Compositions of the Changed Lipid Species

It is known that SLE pathology promotes aberrant lipid metabolism, so these changed lipid species may be related to the disease activity of SLE. Herein, the association between the compositions of the altered lipids and SLEDAI scores was determined using optimal curve fitting (Table 2). The results showed that a significant correlation between the SLEDAI score and 18:0/18:2 dPE (*p* = 0.031) and this lipid specie could explain 22.6% of the variability of the SLEDAI score. A multiple regression was also conducted to predict the SLEDAI score from all the changed lipid species. The analysis suggested that these variables could predict 41.9% of the variability of SLEDAI scores. However, there was no statistical significance (*p* = 0.80) due to the limited sample size in the present study. 

## 4. Discussion

Aberrant lipid metabolism exists in SLE patients due to its pathological factors, including varieties of autoantibodies, elevated oxidative stress, and dysfunction of lipoproteins. Our previous study showed a significant reduction of serum pPE content in SLE patients by MDMS-SL. Moreover, MDMS-SL results strongly substantiated that increased oxidative stress of SLE patients led to the reduced content of pPE species through identification and quantification of their counterpart lipids and metabolites. In other words, the MDMS-SL technology not only revealed the changes of lipids (i.e., triglycerides (TAG) species and oxidative stress-related lipids, such as plasmalogens, lysophospholipids, and HNE species) in SLE, but also uncovered the exact mechanism(s) for the alterations, indicating its powerfulness. In the present study, other important lipids, including phospholipids (i.e., LPC, PI, dPC, and dPE) and sphingolipids (e.g., Cer, and SM) in serum were further determined using the MDMS-SL platform. Their profiles were compared using multivariate analysis and *t* tests between the SLE patients and the normal individuals. dPE (16:0/18:2, 18:0/18:2, 16:0/22:6, 18:0/20:4, and 18:0/22:6), 18:2 LPC, and Cer (N22:0, and N24:1) species with *p* < 0.05 and VIP > 1 were selected and further subjected to examine the association with SLEDAI and proinflammatory cytokines. 

From the results, the major difference between two groups was the profile of dPE species. PE, usually the second most abundant phospholipids in mammalian cells, plays important roles in various cellular functions including signal transduction, vesicle trafficking, and membrane fluidity [37]. Moreover, its profile differs among cell types, organelles, and inner/outer membranes leaflets, and these differences are known to be related with cellular function [38]. In addition, PE species containing polyunsaturated fatty acyls (acids) (PUFAs), such as arachidonic acid, linoleic acid, eicosapentaenoicacid, and docosahexaenicacid (DHA), are major sources of fatty acid (FA)-derived lipid mediators and endocannabinoids [39]. The acyl moieties of PE species synthesized in the de nove pathways are subsequently remodeled by the action of phospholipases and lysophospholipid acyltransferases. This fatty acid remodeling cycle largely contributes to PE diversity and the production of lipid mediators. Usually, PUFAs (e.g., arachidonic acid) are mainly incorporated into glycerophospholipids in the remodeling pathways. However, DHA-containing glycerophospholipids can be synthesized in both the de novo synthesis and remodeling pathways [38]. Therefore, two aspects may be responsible for the changed profiles of dPE in SLE patients. First, arachidonic acid is converted to various eicosanoids to modulate inflammatory events and repair the damage of tissues in SLE patients. Arachidonic acid-containing dPE species are hydrolyzed to release free arachidonic acid, and then varieties of eicosanoids such as prostaglandins and leukotrienes are produced to involve inflammation allergy, and other immune responses in SLE. A series of these reactions resulted in the reduced composition of 18:0–20:4 dPE. Accordingly, the decreased composition of 18:2 FA in serum TAG may facilitate the conversion of 18:2 FA to 20:4 FA [20]. Second, the elevated compositions of dPE (16:0/18:2, 18:0/18:2, 16:0/22:6, and 18:0/22:6) may compensate for the reduced 18:0/20:4 dPE. DHA-containing glycerophospholipids were synthesized in the remodeling pathway to maintain the cellular functions by increasing membrane fluidity and controlling the biophysical properties of the membrane, in addition to serving as the reservoir for producing anti-inflammatory mediators such as resolvins and protectins [40,41,42]. Significant association between dPE species and cytokine IL-10 further support the possibilities.

It is well known that abnormalities in different types of lymphocytes play a crucial role in the pathogenesis of SLE, especially B cells [43]. A large body of evidence indicates that B cells are abnormal in SLE, with the increased number of cells, the elevated calcium flux on signaling through the B-cell receptor, and the high expression of co-stimulatory molecules on B cells [44]. Owing to its multiple pathogenic roles in SLE, understanding the proliferation of B cells is of importance. Cer has a variety of functions in the cell death process depending on its acyl chain length [45] Generally, a shift in Cer composition from C24 to C16 increases vulnerability to cell apoptosis [33]. Therefore, the altered compositions of Cer species may cause the proliferation of lymphocytes cells in SLE. IL-10 can induce mitochondrial hyperpolarization (that is, elevated mitochondrial transmembrane potential) resulting in increased reactive oxygen species [46]. Mitochondrial hyperpolarization was correlated with ATP reduction and necrosis of lupus T cells, which in turn may promote inflammation in patients with SLE [47]. So, the altered compositions of dPE species and of Cer species may have a close relationship with abnormal lymphocytes activation and cell death, which underlie the pathology of SLE. Certainly, further experiments are needed to demonstrate the conclusion.

In summary, the data present here comprehensively described the altered lipid profiles in the serum of SLE patients determined by MDMS-SL. In addition to the alteration in the levels of lipids related to oxidative stress, the results herein suggest that the profiles of some lipids also changed, especially dPE and Cer. Moreover, the altered compositions of the lipids are significantly associated with inflammation and disease activity, contributing to the classification and revealing the underlying mechanism of some symptoms of SLE disease. Therefore, the panel of changed compositions of dPE and Cer species may serve as an additional panel of special biomarkers for early diagnosis and/or prognosis of SLE.

## Figures and Tables

**Table 1:** Demographic and clinical characteristics of the subjects participated in the study * [20].

Parameters	Patients (*N* = 30)	Controls (*N* = 30)
Age (years)	35.0 ± 1.4	35.4 ± 1.5
Gender (% women)	100	100
Disease duration (years)	10.2 ± 1.3	—
Body mass index (Kg/m^2^)	22.6 ± 0.4	23.1 ± 0.5
Anti-dsDNA antibody positivity	20/30	—
Low C3/C4	23/30	—
Proteinuria	13/30	—
SLEDAI	Inactive (0–11): 23/30 Active (>11): 7/30	—

* Values represent the mean ± standard error of the mean (SEM). SLEDAI = systemic lupus erythematosus disease activity index.

**Table 2:** Correlation of SLEDAI score with the compositions of altered lipid species with optimal curve fitting.

Lipid Specie	Equation	R^2^	F	df1	df2	*p*
16:0/18:2 dPE	Quadratic	0.081	1.186	2	27	0.321
18:0/18:2 dPE	Quadratic	0.226	3.951	2	27	0.031
16:0/22:6 dPE	Quadratic	0.087	1.294	2	27	0.291
18:0/20:4 dPE	S	0.056	1.671	1	28	0.207
18:0/22:6 dPE	Cubic	0.115	1.127	3	26	0.356
18:2 LPC	Cubic	0.12	1.848	2	27	0.177
N22:0 Cer	Logarithmic	0.004	0.118	1	28	0.747
N24:1 Cer	Quadratic	0.028	0.392	2	27	0.679

“df” represents degree of freedom.

## Supplementary Materials

The following are available online at http://www.mdpi.com/2218-273X/8/4/105/s1, Table S1: a list of the lipid species used for multivariate analysis.

## Abbreviations

Cer	ceramide
DHA	docosahexaenicacid
HNE	4-hydroxy-2(*E*)-nonenal
ox-LDL	oxidized low-density lipoprotein
MDMS-SL	multi-dimensional mass spectrometry-based shotgun lipidomics technology
PC	phosphatidylcholine
dPC	diacly-phosphatidylcholine
pPC	plasmenylcholine
LPC	lysoPC
PE	phosphatidylethanolamine
aPE	plasmanylethanolamine
dPE	diacyl-phosphatidylethanolamine
pPE	ethanolamine plasmalogen
PI	phosphatidylinositol
LPE	lysoPE
PLS-DA	partial least squares-discriminate analysis
PUFA	polyunsaturated fatty acyl (acid)
SLE	systemic lupus erythematosus
SLEDAI	SLE disease activity index
TAG	triglycerides
