# Supplementary material for: Shotgun Lipidomics Revealed Altered Profiles of Serum Lipids in Systemic Lupus Erythematosus Closely Associated with Disease Activity
Source: Biomolecules. 2018 Oct 3;8(4):105. doi: 10.3390/biom8040105 (PMC6315961; doi:10.3390/biom8040105)
Supplement: Supplementary file 1 [file biomolecules-08-00105-s001.pdf]

Table S1. A list of the lipid species used for multivariate analysis.

	Species	Mean (nmol/mL plasma)		SEM	
		Normal	SLE	Normal	SLE
LPC-1	P16:0	8.93	8.28	0.80	0.86
LPC-2	A16:0	1.46	1.23	0.07	0.11
LPC-3	16:1	10.04	9.53	0.68	0.71
LPC-4	16:0	73.79	71.43	2.51	3.04
LPC-5	P18:0	2.48	2.11	0.23	0.24
LPC-6	18:2	39.36	31.85	2.10	1.19
LPC-7	18:1	14.20	13.63	0.59	0.85
LPC-8	18:0	18.83	16.70	0.83	0.92
LPC-9	20:4	5.98	5.94	0.30	0.44
LPC-10	20:3	1.02	1.05	0.09	0.10
PE-1	D16:0-18:2	1.86	2.45	0.14	0.17
PE-2	D16:0-18:1	0.98	0.85	0.09	0.09
PE-3	D16:1-20:4	1.47	1.11	0.12	0.25
PE-4	D16:0-20:4	2.84	2.88	0.18	0.20
PE-5	D18:1-18:2	1.55	1.83	0.11	0.13
PE-6	D18:0-18:2	4.93	5.74	0.31	0.39
PE-7	D18:0-18:1	1.10	1.00	0.06	0.09
PE-8	D16:0-22:6	3.08	4.58	0.22	0.30
PE-9	D18:1-20:4	2.61	2.46	0.14	0.17
PE-10	D18:0-20:4	10.89	8.31	0.56	0.74
PE-11	D18:0-20:3	0.78	0.87	0.08	0.09
PE-12	D18:1-22:6	0.58	0.63	0.07	0.04
PE-13	D18:0-22:6	2.01	2.69	0.15	0.19
PE-14	D18:0-22:5	0.77	0.76	0.05	0.09
PE-15	D20:0-20:4	0.63	0.25	0.06	0.06
PE-16	D20:0-20:0	0.67	0.44	0.04	0.06
Cer-1	N16:0	0.31	0.29	0.02	0.02
Cer-2	N20:0	0.11	0.11	0.01	0.01
Cer-3	N22:0	0.83	0.78	0.04	0.05
Cer-4	N23:0	0.60	0.56	0.04	0.04
Cer-5	N24:2	0.07	0.07	0.01	0.01
Cer-6	N24:1	0.97	1.38	0.06	0.15
Cer-7	N24:0	2.50	2.54	0.16	0.16
Cer-8	OH_N24:0	0.08	0.05	0.01	0.01
SM-1	N14:0	3.54	3.09	0.24	0.19
SM-2	N15:0	1.92	1.72	0.12	0.11
SM-3	N16:1	10.55	11.26	0.30	0.40
SM-4	N16:0	62.76	68.12	1.73	2.32
SM-5	N17:0	1.01	0.93	0.06	0.07

SM-6	N18:1	5.24	6.18	0.20	0.29
SM-7	N18:0	7.91	9.88	0.28	0.48
SM-8	N20:1	2.90	3.42	0.14	0.23
SM-9	N20:0	5.19	5.52	0.22	0.32
SM-10	N21:0	1.15	0.93	0.09	0.09
SM-11	N22:2	0.84	0.99	0.08	0.14
SM-12	N22:1	9.56	9.50	0.41	0.64
SM-13	N22:0	11.24	10.19	0.39	0.44
SM-14	N23:1	3.33	2.96	0.18	0.21
SM-15	N23:0	3.37	3.11	0.16	0.21
SM-16	N24:3	1.59	1.59	0.11	0.14
SM-17	N24:2	13.42	14.74	0.72	0.89
SM-18	N24:1	22.33	25.41	1.21	2.01
SM-19	N24:0	5.93	5.29	0.24	0.32
PI-1	16:0-18:2	0.84	0.69	0.09	0.09
PI-2	16:0-18:1	0.69	0.64	0.07	0.08
PI-3	16:0-20:4	0.85	0.80	0.09	0.07
PI-4	18:1-18:2	0.79	0.67	0.06	0.07
PI-5	18:0-18:2	3.72	4.09	0.21	0.24
PI-6	18:0-18:1	1.02	0.96	0.08	0.08
PI-7	18:1-20:4	0.66	0.44	0.05	0.04
PI-8	18:0-20:4	10.81	10.02	0.38	0.43
PI-9	18:0-20:3	1.52	1.41	0.12	0.12
PI-10	18:0-22:6	0.40	0.38	0.04	0.04
PI-11	18:0-22:5	0.27	0.25	0.02	0.03
PC-1	D16:1-16:1	1.11	1.19	0.12	0.12
PC-2	D16:1-16:0	2.62	3.12	0.24	0.25
PC-3	D16:0-16:0	5.77	5.14	0.15	0.26
PC-4	D16:1-18:2	4.48	5.03	0.23	0.33
PC-5	D16:0-18:2	269.43	284.08	9.50	11.32
PC-6	D16:0-18:1	73.86	80.19	2.96	5.52
PC-7	D16:0-18:0	1.47	1.02	0.07	0.10
PC-8	A16:0-20:4	11.95	11.19	0.54	0.84
PC-9	D18:2-18:3	6.55	5.14	1.10	0.64
PC-10	D18:2-18:2	98.49	107.30	3.84	6.85
PC-11	D18:1-18:2	65.99	70.43	2.67	3.31
PC-12	D18:0-18:2	147.16	140.61	4.94	5.31
PC-13	D18:0-18:1	11.44	9.25	0.40	0.60
PC-14	D16:0-22:6/D18:2-20:4	38.61	38.00	2.29	2.51
PC-15	D18:1-20:4	15.61	16.04	0.91	1.06
PC-16	D18:2-20:2/D18:0-20:4	54.20	56.41	2.73	3.90

PC-17	D18:0-20:3	6.53	6.82	0.46	0.42
PC-18	D18:0-22:6	9.44	8.52	0.64	0.60
PC-19	D18:0-22:5	1.80	1.66	0.18	0.19
